# Effects of hearing intervention on physical function: A secondary analysis of the ACHIEVE study

**DOI:** 10.1371/journal.pone.0347500

**Published:** 2026-04-29

**Authors:** Jennifer A. Deal, Wuyang Zhang, Kening Jiang, Alison Huang, James Russell Pike, Michelle Arnold, Sheila Burgard, Ziheng (Sally) Chen, Theresa Chisolm, David Couper, Adele M. Goman, Nancy W. Glynn, Theresa Gmelin, Lisa Gravens-Mueller, Kathleen M. Hayden, Christine M. Mitchell, James S. Pankow, Nicholas S. Reed, Victoria Sanchez, Jennifer A. Schrack, Kevin Sullivan, Frank R. Lin, Josef Coresh, on behalf of the ACHIEVE Collaborative Research Group

**Affiliations:** 1 Department of Epidemiology, Johns Hopkins Bloomberg School of Public Health, Baltimore, Maryland, United States of America; 2 Cochlear Center for Hearing and Public Health, Johns Hopkins Bloomberg School of Public Health, Baltimore, Maryland, United States of America; 3 Department of Otolaryngology-Head and Neck Surgery, Johns Hopkins School of Medicine, Baltimore, Maryland, United States of America; 4 Amplifon SpA, Milan, Italy; 5 Department of Communication Sciences & Disorders, College of Behavioral & Community Science, University of South Florida, Tampa, Florida, United States of America; 6 Department of Biostatistics, Gillings School of Global Public Health, University of North Carolina, Chapel Hill, North Carolina, United States of America; 7 School of Health and Social Care, Edinburgh Napier University, Edinburgh, United Kingdom; 8 Department of Epidemiology, University of Pittsburgh School of Public Health, Pittsburgh, Pennsylvania, United States of America; 9 Department of Social Sciences and Health Policy, Wake Forest University School of Medicine, Winston-Salem, North Carolina, United States of America; 10 Division of Epidemiology and Community Health, University of Minnesota School of Public Health, Minneapolis, Minnesota, United States of America; 11 Department of Otolaryngology-Head & Neck Surgery, Morsani College of Medicine, University of South Florida, Tampa, Florida, United States of America; 12 Center on Aging and Health, Johns Hopkins University, Baltimore, Maryland, United States of America; 13 The MIND Center, University of Mississippi Medical Center, Jackson, Mississippi, United States of America; 14 Optimal Aging Institute, New York University Grossman School of Medicine, New York, New York, United States of America; Fondazione Don Carlo Gnocchi, ITALY

## Abstract

**Objective:**

We tested the effect of the hearing intervention vs. health education control (1:1 randomization) on 3-year physical function decline, a secondary analysis of the ACHIEVE randomized trial (ClinicalTrials.gov Identifier: NCT03243422).

**Methods:**

Pre-specified outcomes included the Short Physical Performance Battery (SPPB) [total score and components] and grip strength. Intervention effects were modeled using linear mixed models following a pre-specified statistical analysis plan.

**Results:**

In 956 participants (mean 76.3 years, 53% female, 12% Black race), hearing intervention did not impact change in SPPB (difference comparing intervention to control = 0.00 standard deviation [SD] units, 95% confidence interval [CI]:-0.14, 0.14) or grip strength (difference = 0.01 SD, 95% CI: −0.06, 0.08). However, treatment effects varied by recruitment cohort; although findings were not statistically significant, they suggest a clinically meaningful benefit (slower decline) in participants with faster rates of cognitive decline.

**Conclusions:**

These secondary results have direct relevance for inclusion in systematic reviews and meta-analyses. Future research should be designed to test whether hearing intervention can reduce short-term declines in physical function, particularly among those experiencing cognitive decline. Continuing follow-up of the ACHIEVE study participants will yield insights into longer-term (>3 years) effects of intervention.

## Introduction

Hearing loss is an underrecognized risk factor for physical function decline in older adults. Observational studies suggest an association between hearing loss and gait speed [1–3] and poorer lower mobility function [3–8]. These associations could be explained through a common underlying pathology, such as concomitant vestibular dysfunction [9–10]. Alternatively, hearing loss may cause physical function decline through mechanisms including accelerated cognitive decline, increased cognitive load, or reduced auditory awareness of the environment [11–12].

Few studies have investigated if hearing aids mitigate the association between hearing loss and physical function decline, although clinic-based studies suggest that they improve postural stability and static balance [13–16]. The Aging and Cognitive Health Evaluation in Elders (ACHIEVE) study is a multicenter, parallel-group, unmasked randomized controlled trial that tested the efficacy of a best-practices hearing intervention vs. health education control (1:1 allocation ratio) on 3-year cognitive decline (primary outcome) in older adults aged 70–84 years with untreated hearing loss and without substantial cognitive impairment (ClinicalTrials.gov Identifier: NCT03243422) [17–18]. The primary outcome of the ACHIEVE study was 3-year change in global cognitive function. Here, in a secondary analysis, we report on the effect of the hearing intervention vs. control on 3-year change in physical function, a pre-specified exploratory outcome of the trial. We hypothesized that hearing intervention reduces decline in Short Physical Performance Battery (SPPB) [19] performance but does not affect grip strength. Given impacts of hearing on balance [13–16], we anticipated that any intervention effects are driven by reduced balance declines.

## Materials and methods

### Trial design

Multicenter, parallel-group, unmasked, active-controlled randomized trial with a 1:1 allocation ratio.

### Participants, eligibility criteria, and settings

The ACHIEVE study is based within the Atherosclerosis Risk in Communities (ARIC) study, an ongoing longitudinal study in four US field sites (Forsyth County, NC; Jackson, MS; Minneapolis, MN; and Washington County, MD) of 15,792 adults aged 45–64 years were recruited from a random community sample in 1987–89 (S1 File) [20]. Participants continue to be followed as part of the ARIC-Neurocognitive Study (ARIC-NCS) [20–21].

ACHIEVE participants (N = 977) were recruited from the four ARIC sites between 2018–2019; 238 participants were from ARIC-NCS, and 739 participants were de novo volunteers from the community. Inclusion criteria were: 70–84 years old; community-dwelling; audiometric hearing loss [better-hearing ear pure-tone average (PTA) ≥30 and <70 dB hearing level (dB HL)]; Mini-Mental State Exam ≥23 for high school degree or less, and ≥25 for some college or more; Word Recognition in Quiet score ≥60% correct (better-hearing ear); fluent English-speaker; and remaining in the area during the study. Exclusion criteria were: self-reported difficulty in ≥2 activities of daily living [22]; prior dementia diagnosis; presenting vision worse than 20/63 (~14-point font) with usual correction [23]; medical contraindication to hearing treatment; untreatable conductive hearing impairment; unwillingness to regularly wear hearing aids; and self-reported hearing aid use in the past year [17,18,24]. We excluded participants missing baseline SPPB (n = 10), grip strength (n = 5), education (n = 1), and BMI (n = 5). Our analytic sample was n = 956.

The recruitment process for the trial has been previously described in detail [24]. A soft-launch of recruitment efforts began at a limited number of study sites in November 2017 and full-scale efforts at all sites in January-February 2018. The original target sample size (N = 850) was achieved in July 2019. After interim Data Safety and Monitoring Board (DSMB) analysis of drop-in, drop-out and missing data, and prior to recruitment closure, with National Institute of Aging approval, investigators were authorized to extend the recruitment window, which closed on October 27, 2019 with a final sample of N = 977. The trial concluded as scheduled at the end of 3 years. The Institutional Review Boards (IRBs) at each collaborating site, including the 4 field centers and the coordinating center (University of Mississippi Medical Center IRB, University of Minnesota IRB, Wake Forest University IRB, Johns Hopkins Bloomberg School of Public Health IRB, University of North Carolina IRB) approved the trial, and participants provided written informed consent. An independent data and safety monitoring board oversaw study progress, adverse events, and changes to the study protocol and statistical analysis plan. We followed EQUATOR reporting guidelines (S2 File) [25].

### Interventions

For the hearing intervention, participants completed four 1-hour sessions with an audiologist spaced every 1–3 weeks post-randomization. Intervention included bilateral hearing aids fitted to prescriptive targets using real-ear measures, paired with hearing-assistive technologies (e.g., devices to stream smartphones and television), systematic orientation and instruction in device use, and provision of hearing toolkit materials for self-management and communication strategies [26–27]. Re-instruction was provided during booster visits every 6 months [17].

For the health education control intervention, participants completed four 1-hour sessions with a certified health educator spaced every 1–3 weeks post-randomization with subsequent booster sessions every 6 months. The intervention included administration of the 10 Keys^TM^ to Healthy Aging program [28], an evidence-based, interactive, health education program for older adults on topics relevant to chronic disease and disability prevention [17].

### Outcome measures

Physical function, including the SPPB and grip strength, were pre-specified secondary outcomes of the trial. SPPB [19] and grip strength were measured at baseline and each annual in-person follow-up visit. The SPPB is a series of physical performance tests (chair stands, balance, 4-meter walk) to assess lower extremity function in older adults that predicts mortality [29], falls [30], hospitalizations [31] and incident disability [19,32]. For chair stands, participants were asked to fold arms over chest and rise to standing from a seated position in a chair. If able to complete one, participants were timed as they completed five in a row, as quickly as possible without stopping, keeping arms folded over chest. Standing static balance scores are based on the participant’s ability to hold 3 balance tasks (side-by-side, semi-tandem, full tandem) for 10 seconds each. To maximize time allotment for testing, participants started with feet in a semi-tandem position. If able to hold for the full 10 seconds, it was assumed that they would also be able to hold the easier side-by-side position and were scored as having completed it. They then attempted the full tandem stand. If participants were unable to hold the semi-tandem stand, they attempted the side-by-side position and received a score of 0 seconds for the full tandem position.

Standardized SPPB scoring is available, ranging from 0 to 12 [19,33]. However, given that our population was high-functioning, and that ceiling effects in high-functioning populations can lead to biased effect estimates [34], for analysis, we rescaled SPPB scores according to published guidelines developed in the Health Aging and Body Composition study [35]. To account for non-normality, we converted testing times to rates for chair stands (chair stands/s) and 4-meter walk (m/s), assigning a score of 0 when a participant was not able to perform the test. For standing balance, we summed the time for the 3 balance positions. Component scores were then divided by the maximal performance possible based on external data from other studies (1 chair stand/s; 2 m/s walk time; 30 seconds for balance) to derive 3 component ratio scores, each ranging from 0 to 1. We then summed the component ratio scores to get a continuous rescaled SPPB summary score ranging from 0 to 3, with higher scores indicating better performance.

Grip strength was measured in kg with a Jamar handheld dynamometer in the dominant hand. After one practice trial, participants completed two trials, squeezing as hard as possible, with a 15–20 second rest between trials. The two trials were averaged for analysis, modeled continuously.

Component scores were modeled as speed for chair stands (number of chairs stands/s) and the 4-meter walk (m/s), with higher scores indicating faster completion rates. Because time to complete the standing balance trials was heavily skewed, we modeled balance as a binary variable taking a value of 0 if unable to hold all 3 positions for the full time, and 1 if able.

We standardized continuous outcome scores to baseline values by subtracting baseline means and dividing by baseline standard deviation (SD) in our analytic sample. Baseline distributions for outcome measures are therefore mean zero, SD of one, with subsequent values representing change relative to baseline.

### Minimal detection effect size calculations

Given that physical function was a secondary outcome, the ACHIEVE study was not powered to detect differences in rates of SPPB change over time. To guide the interpretation of the results of the present study, we conducted post-hoc estimation of the minimal detection effect size estimates. These estimates suggest, at the alpha = 0.05 level, our study can detect a mean difference in SPPB score change comparing intervention to control of at least 0.36 points with 80% power and 0.42 points with 90% power.

### Interim analyses and stopping guidelines

There were no interim analyses for efficacy.

### Randomization (random number generation, allocation concealment, implementation)

Randomization was determined by an allocation schedule developed by the Data Coordinating Center at the University of North Carolina (Chapel Hill, NC, USA) and completed within the CDART, the web-based data management system. Participants were randomized in a 1:1 ratio to either the hearing or the control intervention To ensure balance between the treatment groups, participants were randomized in permuted order blocks of varying sizes within strata defined by severity of hearing loss, defined as mild (PTA ≥ 30dB and <40dB) or moderate (PTA ≥ 40 dB and <70dB), recruitment source (ARIC or de novo participant), and by field site. Block size was not revealed to field center staff as this would have allowed them to determine the final treatment assignment of a block before ascertaining eligibility and obtaining consent. [17].

### Masking

Participants and study staff collecting outcome data were unmasked to treatment assignment. Participants were masked to study hypothesis. ACHIEVE investigators and staff (except coordinating center staff and one statistician) were masked to trial data until the primary findings were reported [17].

### Statistical analysis

**Fig 1:**
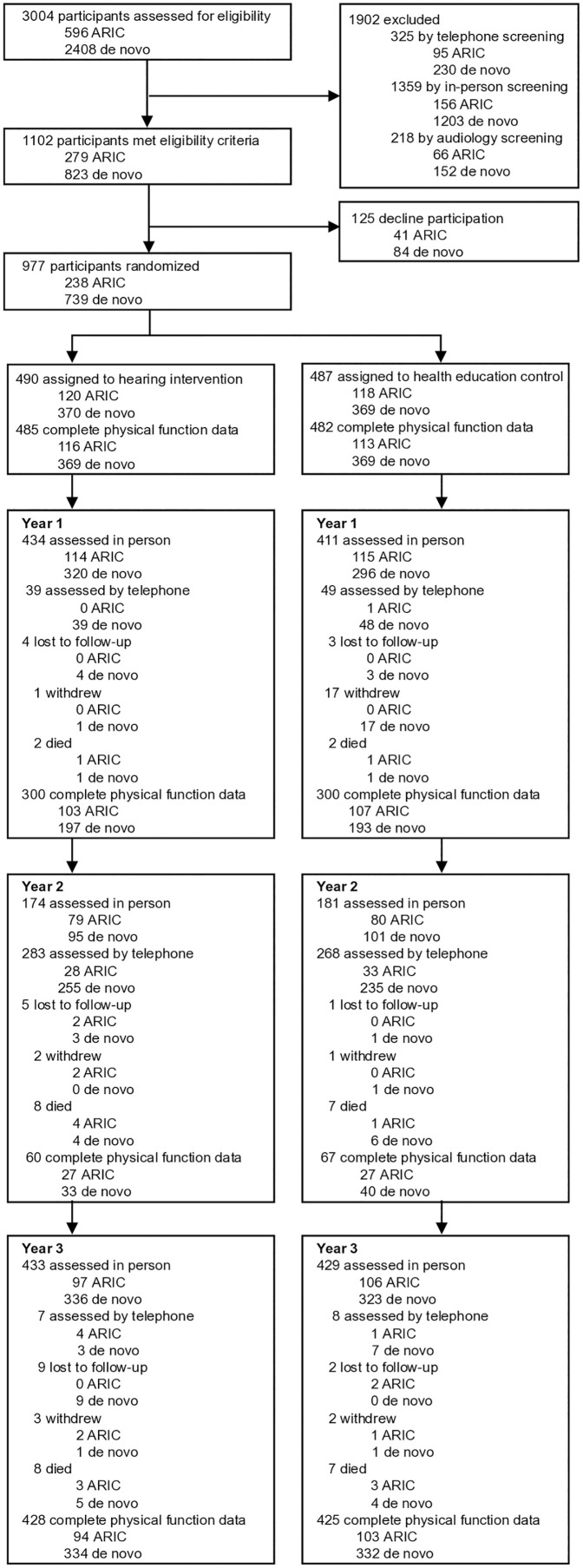
Consort Flow Diagram for Analysis of Physical Function, Aging and Cognitive Health Evaluation in Elders (ACHIEVE) Study, 2018−19 to 2021−22. Abbreviations: ARIC, Atherosclerosis Risk in Communities; PF, Physical Function. Participants must have completed all components of the physical function battery (chair stands, balance, walking speed, grip strength) to have complete physical function data.

Participant characteristics were compared by intervention assignment and recruitment source. Our primary analysis was intention-to-treat and restricted to available cases, estimating the effect of the hearing intervention on 3-year change in physical function outcomes (rescaled SPPB score and grip strength) using two-level linear mixed effects models with an unstructured covariance matrix for the random effects (intercepts and slopes). We restricted to data from baseline and Year 3 given large amounts of missing outcome data during the intervening years due to COVID-19 pandemic shutdowns of the study clinics (Fig 1). Conversions to account for non-normality of some outcomes are described in the Outcome Measures section of the Methods. Time was modeled continuously, and an interaction term between time and intervention assignment was the regression coefficient used to test if the rate of change in the outcome differed by intervention assignment. Restricted maximum likelihood with a Kenward-Roger correction was used to generate parameter estimates and 95% confidence intervals (CI); these methods were chosen over others (e.g., maximum likelihood methods) as they can reduce bias in smaller sample sizes [36]. To control for possible residual confounding, models adjusted for baseline age, sex, race, field site, education, recruitment source, BMI, hearing loss severity, and interaction terms between time and all covariates except education. Participants self-reported age (years, modeled continuously), sex (male, female), educational attainment (less than high school, high school or equivalent, greater than high school) and race (Black, White, Asian, American Indian or Alaska Native) at baseline. Recruitment source was recorded as ARIC-NCS or de novo. Body mass index (BMI; kg/m^2^) was calculated using measured height and weight and modeled continuously. Audiometric thresholds at 0.5, 1, 2, and 4 kHz were averaged, and a binary variable was created to indicate greater hearing loss severity (PTA ≥ 40 dB HL vs. < 40 dB HL) in the better-hearing ear. Model fit was adequate and assumptions for linearity met as assessed using standard fit indices, including the AIC and BIC and by visualizing the measures and model residuals over time. For grip strength, given documented sex differences in cutpoints for clinically relevant weakness [37], we included an interaction term between sex and baseline grip strength, and a 3-way interaction between sex, intervention assignment, and time, to estimate the hearing intervention effect by sex.

In additional analyses, we modeled the SPPB components separately. Linear mixed models to estimate rate differences by intervention group for chair stands and walking speed are the same as those described above, including adjustment covariates. We estimated the marginal odds ratio of inability to hold all 3 positions over follow-up using Generalized Estimating Equations (GEE) [38] with an unstructured correlation matrix and robust standard errors. Adjustment factors were the same as above.

Given that findings for the primary ACHIEVE trial outcome (3-year cognitive decline) differed by recruitment source [17], and hypotheses that the effect of hearing loss on balance may be stronger in individuals with poorer cognition [39], we conducted a sensitivity analysis that was pre-specified in the statistical analysis plan, which was registered at ClinicalTrials.gov before the unmasking of trial data, and re-ran the analysis, stratifying by recruitment source.

We conducted 5 additional sensitivity analyses. Under a missing at random assumption, missing outcome and covariate information was imputed using multiple imputations by chained equations for participants who were alive at the time of the study visit. We re-ran models, including available Year 1 outcome data. We also modeled the original SPPB score, to allow comparisons with published literature. We conducted a per-protocol analysis excluding study participants who did not follow their assigned intervention. Finally, we estimated the complier average causal effect (CACE) of hearing intervention among participants assigned to hearing intervention. Methodological details for sensitivity analyses are provided in supplemental materials (S3 File).

Because the trial was not powered to detect intervention differences for this pre-specified exploratory outcome, we focused on patterns of associations for hypothesis-generation instead of hypothesis-testing for statistical significance. All analyses were conducted using Stata 18.0 (StataCorp, College Station, TX).

## Results

Overall, mean participant age was 76.3 years, 53% were female, 12% self-reported Black race and 54% had a Bachelor’s degree or higher (Table 1). In our analytic sample, 479 participants were randomized to the hearing intervention, with the remaining 477 to the health education control. Intervention groups were balanced with respect to measured covariates (Table 1). Retention over 3 years was high, with 87% completing in-person assessments at Year 3 for both the intervention and control arms (Fig 1) [17]. Seventy-six participants (17%) randomized to the control intervention obtained hearing aids during the study period. Only 10 individuals (2%) randomized to the hearing intervention did not complete the intervention per study protocol [17]. Adverse events (otitis externa, cerumen impaction or ear foreign body requiring removal by a physician, and all-cause mortality) were monitored by study investigators and the DSMB throughout the study. No adverse events were unexpected or judged to be related to study participation [17].

**Table 1:** Distributions of Baseline Participant Characteristics by Randomized Intervention Assignment and Recruitment Source, Aging and Cognitive Health Evaluation in Elders (ACHIEVE) Study, 2018−19.

	Overall	Total Cohort (N = 956)		ARIC (n = 226)		De novo (n = 730)	
		Control	Intervention	Control	Intervention	Control	Intervention
	N = 956	n = 477	n = 479	n = 112	n = 114	n = 365	n = 365
Age, mean (SD), y	76.3 (3.9)	76.5 (4.0)	76.0 (3.9)	78.0 (2.9)	78.7 (2.8)	76.0 (4.2)	75.2 (3.8)
Body mass index, mean (SD), kg/m2	29.0 (5.5)	29.0 (5.6)	29.0 (5.4)	28.8 (6.0)	29.6 (4.7)	29.0 (5.5)	28.8 (5.6)
Self-reported female sex, N(%)	509 (53.2)	252 (52.8)	257 (53.7)	69 (61.6)	70 (61.4)	183 (50.1)	187 (51.2)
Self-identified race, N(%)							
Black	111 (11.6)	59 (12.4)	52 (10.9)	35 (31.2)	32 (28.1)	24 (6.6)	20 (5.5)
White	838 (87.7)	414 (86.8)	424 (88.5)	77 (68.8)	81 (71.1)	337 (92.3)	343 (94.0)
Asian	6 (0.6)	4 (0.8)	2 (0.4)	0 (0.0)	1 (0.9)	4 (1.1)	1 (0.3)
American Indian or Alaska Native	1 (0.1)	0 (0.0)	1 (0.2)	0 (0.0)	0 (0.0)	0 (0.0)	1 (0.3)
Field site, N(%)							
Forsyth County, North Carolina	228 (23.8)	114 (23.9)	114 (23.8)	29 (25.9)	30 (26.3)	85 (23.3)	84 (23.0)
Jackson, Mississippi	242 (25.3)	123 (25.8)	119 (24.8)	33 (29.5)	29 (25.4)	90 (24.7)	90 (24.7)
Minneapolis, Minnesota	235 (24.6)	115 (24.1)	120 (25.1)	21 (18.8)	21 (18.4)	94 (25.8)	99 (27.1)
Washington County, Maryland	251 (26.3)	125 (26.2)	126 (26.3)	29 (25.9)	34 (29.8)	96 (26.3)	92 (25.2)
Educational attainment, N(%)							
Less than high school	36 (3.8)	18 (3.8)	18 (3.8)	10 (8.9)	11 (9.6)	8 (2.2)	7 (1.9)
High school or equivalent	402 (42.1)	205 (43.0)	197 (41.1)	43 (38.4)	44 (38.6)	162 (44.4)	153 (41.9)
College, graduate, or professional school	518 (54.2)	254 (53.2)	264 (55.1)	59 (52.7)	59 (51.8)	195 (53.4)	205 (56.2)
Better-ear PTA ≥ 40 dB HL, N(%)	415 (43.4)	211 (44.2)	204 (42.6)	46 (41.1)	46 (40.4)	165 (45.2)	158 (43.3)

Abbreviations: ARIC, Atherosclerosis Risk in Communities Study; dB HL, decibel hearing level; PTA, pure-tone average; SD, standard deviation

Compared to participants recruited de novo from the community, participants recruited from ARIC-NCS were more likely to be older, self-identify as Black race and have lower educational attainment, although covariate balance by intervention assignment was maintained within each recruitment cohort (Table 1). Mean hearing levels were similar across recruitment cohorts. Drop-in (randomized to control but obtained hearing aids) was more common among participants from the de novo cohort (19% vs. 8% from ARIC-NCS) [17].

Baseline total SPPB scores were, on average, in the intermediate to high functioning range, with a mean total score of 10.0 (SD 2.0) in the total population (S4 Table). Baseline scores were similar across intervention arms but were slightly lower in individuals recruited from ARIC-NCS (9.4 vs. 10.1 in the de novo cohort). Overall, mean SPPB scores declined over time to 9.1 (SD 2.6) by Year 3. Rates of decline were similar by recruitment cohort. Mean grip strength at baseline was 35.5 kg (SD 8.1) in males and 21.4 kg (SD 5.1) in females (S4 Table). Baseline performance for the SPPB components were similar by intervention group, but slightly worse in the ARIC-NCS vs. de novo cohort (S5 Table).

**Fig 2:**
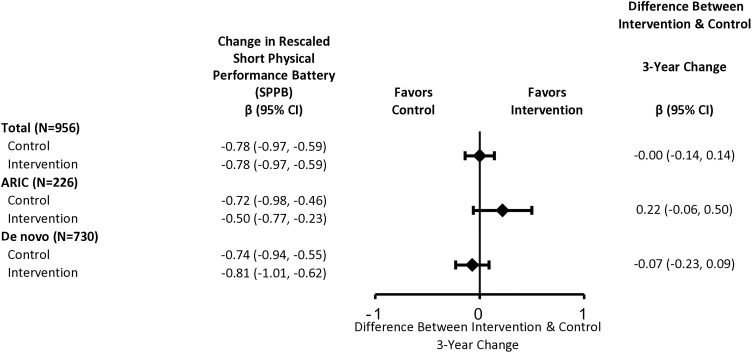
Multivariable-adjusted 3-Year Estimated Changes in Rescaled Short Physical Performance Battery (SPPB) Scores by Randomized Intervention Assignment and Recruitment Source, The Aging and Cognitive Health Evaluation in Elders (ACHIEVE) Study, 2018-22. **Abbreviations:** ARIC: The Atherosclerosis Risk in Communities Study; CI: confidence interval. Estimates in the total cohort were obtained using linear mixed effects models with random intercepts, random slopes, and unstructured covariance. Models included treatment, time since baseline, and an interaction term between time and treatment. Models were adjusted for age, sex, race, field site, education, recruitment source, body mass index, and pure-tone average. The rescaled SPPB score includes chair stand rate (chair stands/second), standing balance (total time in seconds participants held in side-by-side, semi-tandem, full-tandem positions) and 4-meter walking speed (meters/second). For each component, participants’ performance was divided by maximum performance (1 chair stand/second; 30 seconds; 2 meters/second) and was thus converted to a ratio ranging from 0-1. Total rescaled SPPB scores range from 0-3; higher scores indicate better performance. The rescaled SPPB score was standardized for analysis by subtracting baseline mean and then dividing by baseline standard deviation. Estimates by recruitment source were obtained using linear mixed effects models with random intercepts, random slopes, and unstructured covariance. Models included treatment, recruitment source, time since baseline, an interaction term between treatment and recruitment source, an interaction term between time and treatment, an interaction term between time and recruitment source, and a three-way interaction term between time, treatment, and recruitment source. Models were adjusted for age, sex, race, field site, education, body mass index, and pure-tone average.

**Fig 3:**
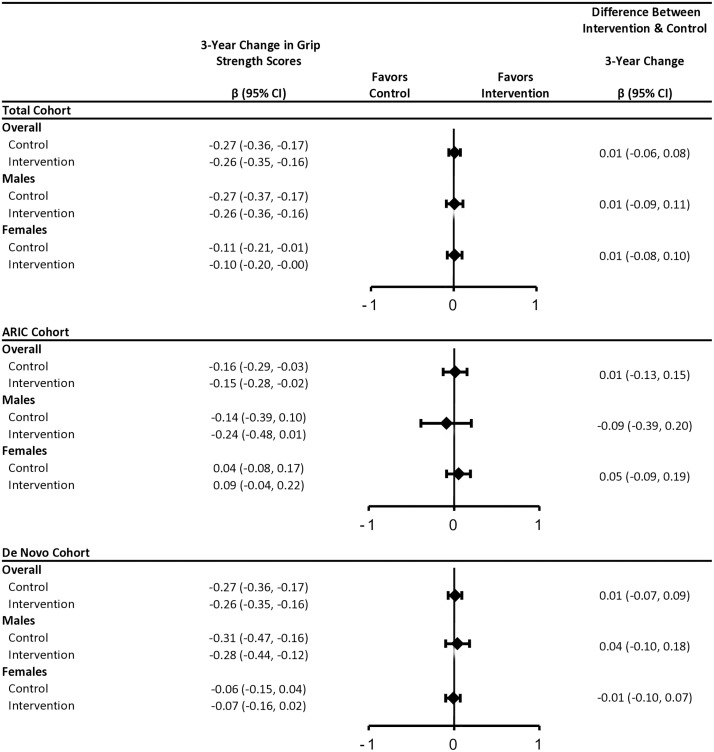
Multivariable-adjusted 3-Year Estimated Changes in Grip Strength by Randomized Intervention Assignment, Recruitment Source and Biological Sex, The Aging and Cognitive Health Evaluation in Elders (ACHIEVE) Study, 2018−22. Abbreviations: ARIC: The Atherosclerosis Risk in Communities Study; CI: confidence interval. Estimates in the total cohort were obtained using linear mixed effects models with random intercepts, random slopes, and unstructured covariance. Models included treatment group, time since baseline, biological sex, an interaction term between sex and baseline grip strength, and a 3-way interaction between sex, treatment group, and time. Models were adjusted for age, race, field site, education, recruitment source, body mass index, and pure-tone average. Grip strength was assessed by dynamometer, and the strength in kilograms obtained from two trials were averaged. Grip strength was standardized by subtracting baseline mean and then dividing by baseline standard deviation. Estimates by recruitment source were obtained using sex-stratified linear mixed effects models with random intercepts, random slopes, and unstructured covariance. Models included treatment, time since baseline, and an interaction term between time and treatment. Models were adjusted for age, race, field site, education, body mass index, and pure-tone average.

In the total ACHIEVE population, intervention did not impact rates of decline in SPPB or grip strength (Fig 2, S6 Table). Effect estimates differed by cohort (intervention effect = 0.22 SD [95% CI: −0.06, 0.50] in ARIC-NCS vs. −0.07 SD [95% CI: −0.23, 0.09] in de novo), but confidence intervals were wide (Fig 2, S6 Table). Rates of decline in grip strength were faster in males than females, with no intervention effect (Fig 3, S6 Table).

**Fig 4:**
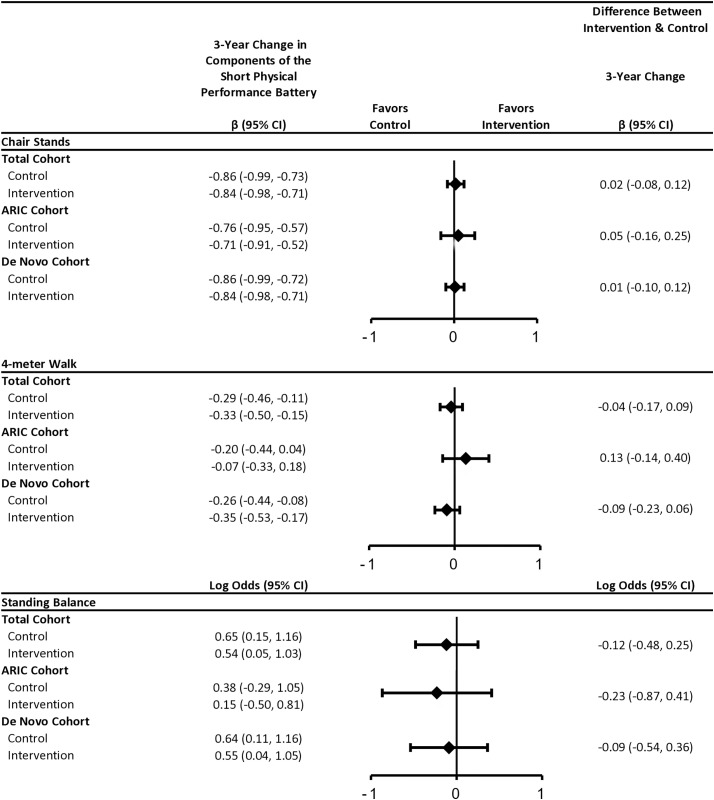
Multivariable-adjusted 3-Year Estimated Changes in Short Physical Performance Battery (SPPB) Component Scores (Chair Stands, 4-meter Walk, Standing Balance) by Randomized Intervention Assignment and Recruitment Source, The Aging and Cognitive Health Evaluation in Elders (ACHIEVE) Study, 2018−22. Abbreviations: ARIC: The Atherosclerosis Risk in Communities Study; CI: confidence interval. Estimates in the total cohort were obtained using linear mixed effects models with random intercepts, random slopes, and unstructured covariance. Models included treatment, time since baseline, and an interaction term between time and treatment. Models were adjusted for age, sex, race, field site, education, recruitment source, body mass index, and pure-tone average. Chair stand rates (number of chair stands/second) and 4-meter walk speeds (m/s) were standardized for analysis by subtracting baseline mean and then dividing by baseline standard deviation. Standing balance was modeled as a binary variable (no vs. yes [reference]), as the ability to hold all three positions (side-by-side, semi-tandem, full-tandem) for the full time. Estimates by recruitment source were obtained using linear mixed effects models with random intercepts, random slopes, and unstructured covariance. Models included treatment, recruitment source, time since baseline, an interaction term between treatment and recruitment source, an interaction term between time and treatment, an interaction term between time and recruitment source, and a three-way interaction term between time, treatment, and recruitment source. Models were adjusted for age, sex, race, field site, education, body mass index, and pure-tone average.

Overall, hearing intervention did not impact change in SPPB component scores (Fig 4, S7 Table). Treatment effects differed by cohort for 4-meter walk speed, but wide confidence intervals preclude conclusions that treatment slowed rates compared to control. No difference in effect estimates were observed by cohort for chair stands or standing balance.

Results were similar in sensitivity analyses, including multiple imputation (S8, S9 Tables), addition of Year 1 outcome data (S10, S11 Tables), use of the original (non-transformed) SPPB (S12 Table), per-protocol (S13 Fig), and CACE analyses (S14 Fig).

## Discussion

In this secondary analysis of a randomized trial testing the effect of hearing intervention vs. health education control in older adults aged 70–84 years with untreated mild-to-moderate hearing loss and without substantial cognitive impairment, we found no efficacy signal for a benefit of treatment on lower extremity function. The magnitude and direction of the treatment effect varied by recruitment cohort, with a suggestion of a benefit (slower decline) in those participants recruited from an ongoing prospective cohort study that was not observed in the cohort of healthy participants newly recruited for the study. The estimated effect (0.22 SD slower decline over 3 years) equates to 0.53 points on the SPPB, suggestive of a clinically significant change [40], although a wide confidence interval precludes definitive conclusions. However, the trial was not powered to detect intervention differences in rates of physical function decline. Given the primary trial findings of reduced rates of cognitive decline among those with increased risk for cognitive decline (those in the ARIC-NCS cohort) [17], prior hypotheses that hearing treatment may have stronger effects on physical function among those with greater cognitive impairment [39], and strong mechanistic hypotheses for why hearing treatment may impact physical function, our results are not inconsistent with possible effect and support future studies designed to explicitly test whether hearing treatment can slow physical function decline in older adults. We highlight that our findings are based only on one study sample and future studies should enroll larger sample sizes specifically powered to address this research question, with participants recruited other geographical areas, in order to confirm the possible efficacy of the hearing intervention to delay physical function decline in older adults. Our findings also contribute to future systematic reviews and meta-analyses designed to address this question.

Prior observational studies report an association between hearing loss and lower extremity function [1–8] but few have tested the impact of hearing aid use, with mixed results [5–6]. In 355 participants with hearing loss (4-frequency PTA ≥ 25 dB HL, mean age 69 years, 55% female), hearing aid users (28%) vs. non-users walked 400 meters 24 seconds faster at baseline, but use did not impact rates of 6-year decline [6]. Hearing aid use was not associated with baseline or longitudinal differences in walking endurance or SPPB performance in a study of 811 men and women (mean age 79 years) with moderate or greater hearing loss [5]. A recent review of laboratory-based studies suggests that hearing loss may have a stronger effect on balance in individuals with cognitive impairment, but more studies are needed [36]. As physical function was an exploratory outcome of the ACHIEVE trial, inference from our study is limited. However, given the scant number of studies in this area, we believe our study is a novel, and needed, contribution to the literature.

Non-causal pathways linking hearing loss to decreased physical function include a shared underlying pathology, such as concomitant vestibular dysfunction [9–10]. We did not measure vestibular function and so are unable to evaluate its impact in our study, however, given randomization, it is likely that it was balanced between our intervention groups. One potential causal pathway by which hearing loss may lead to physical function decline is through reduced auditory awareness of the sound cues in the environment that would typically help with orientation [11,39]. Hearing loss may also potentially lead to physical function decline through increased cognitive load (increased cortical processing effort required to understand a degraded auditory signal) and decline [6,8]. Perhaps due to an interplay of auditory cues and increased cognitive load, laboratory-based studies suggest that unaided individuals with hearing loss may have less ability to maintain postural stability than individuals using hearing aids, particularly in the presence of other challenges decreasing sensory awareness, including white noise [13,16] and vestibular dysfunction [15]. Therefore, we expected to see strongest effects comparing intervention to control for balance in our study. However, treatment effects differed by cohort for 4-meter walk speed, not standing balance. Given that only 20% of our population were unable to hold all 3 balance stances for the full time, it may be that our measure did not have the variability needed to detect a difference. Future studies should consider alternative balance tasks that are more challenging but may be safely tested in this population.

### Limitations

The trial was not powered to detect intervention differences on physical function decline and so our results should be considered hypothesis-generating on their own, although they may contribute to future systematic reviews and meta-analyses. Although all participants completed the baseline visit, the COVID-19 shutdown resulted in missing outcome data, particularly in Years 1 and 2. Results were unchanged in sensitivity analysis using multiple imputation. Given that missingness was due to study visit disruptions rather than participant characteristics, the mechanism for missingness could plausibly be considered missing at random (MAR) conditional on observed covariates. However, we cannot rule out the possibility that missingness was related to unmeasured factors. We may have been lacking strong auxiliary variables related to physical function and to missingness, reducing the potential efficiency gains typically observed under MAR assumptions. Participants are from four US study sites and primarily self-identified White or Black race, and so generalizability of study findings may be limited to other geographic locations and race/ethnicity groups. Participants were not masked to intervention assignment, but physical function was measured objectively in our study, reducing the possibility of bias in outcome reporting by treatment group.

## Conclusions

These exploratory findings support future research designed to test whether non-pharmacologic hearing intervention can reduce short-term declines in physical function, particularly among those who may also be experiencing cognitive decline. Continuing follow-up of the ACHIEVE cohort will yield insights into longer-term (>3 years) effects of intervention on physical function decline.

## Supporting information

S1 File ACHIEVE Protocol. (PDF)

S2 File Consort Checklist. (PDF)

S3 File Appendix. Supplemental Methods.
(PDF)

S1 Table Distributions of Baseline and Follow-up of Total Short Physical Performance Battery (SPPB) and Grip Strength Scores, by Randomized Intervention Assignment, Recruitment Source, and Sex (Grip Strength Only), The Aging and Cognitive Health Evaluation in Elders (ACHIEVE) study, N = 956, 2018−22. (PDF)

S2 Table Distributions of Baseline and Follow-up Short Physical Performance Battery (SPPB) Component Scores (Original and Transformed), by Randomized Intervention Assignment and Recruitment Source, The Aging and Cognitive Health Evaluation in Elders (ACHIEVE) study, N = 956, 2018−22. (PDF)

S3 Table Multivariable-adjusted 3-Year Estimated Changes in Rescaled Short Physical Performance Battery (SPPB) and Grip Strength Scores by Randomized Intervention Assignment, Recruitment Source and Sex, The Aging and Cognitive Health Evaluation in Elders (ACHIEVE) study, N = 956, 2018−22. (PDF)

S4 Table Multivariable-adjusted 3-Year Estimated Changes in Components of the Short Physical Performance Battery (Chair Stands, 4-meter Walk, Balance) by Randomized Intervention Assignment and Recruitment Source, The Aging and Cognitive Health Evaluation in Elders (ACHIEVE) study, N = 956, 2018−22. (PDF)

S5 Table Sensitivity Analysis with Multiple Imputation to Impute Missing Data: Multivariable-adjusted 3-Year Estimated Changes in Rescaled Short Physical Performance Battery (SPPB) Scores and Grip Strength by Randomized Intervention Assignment and Recruitment Source, The Aging and Cognitive Health Evaluation in Elders (ACHIEVE) study, N = 977, 2018−22. (PDF)

S6 Table Sensitivity Analysis with Multiple Imputation to Impute Missing Data: Multivariable-adjusted 3-Year Estimated Changes in Components of the Short Physical Performance Battery (Chair Stands, 4-meter Walk, Balance) by Randomized Intervention Assignment and Recruitment Source, The Aging and Cognitive Health Evaluation in Elders (ACHIEVE) study, N = 977, 2018−22. (PDF)

S7 Table Sensitivity Analysis with Models Including Year 1 Data, in Addition to Baseline and Year 3: Multivariable-adjusted 3-year Estimated Changes in Rescaled Short Physical Performance Battery (SPPB) Scores and Grip Strength by Randomized Intervention Assignment and Recruitment Source, The Aging and Cognitive Health Evaluation in Elders (ACHIEVE) study, N = 956, 2018−22. (PDF)

S8 Table Sensitivity Analysis with Models Including Year 1 Data, in Addition to Baseline and Year 3: Multivariable-adjusted 3-year Estimated Changes in Components of the Short Physical Performance Battery (Chair Stands, 4-meter Walk, Balance) by Randomized Intervention Assignment and Recruitment Source, The Aging and Cognitive Health Evaluation in Elders (ACHIEVE) study, N = 956, 2018−22. (PDF)

S9 Table Sensitivity Analysis Using the Original SPPB Score: Multivariable-adjusted 3-year Estimated Changes in Short Physical Performance Battery (SPPB) Scores by Randomized Intervention Assignment and Recruitment Source, The Aging and Cognitive Health Evaluation in Elders (ACHIEVE) study, N = 954, 2018−22. (PDF)

S1 Fig Per-protocol Sensitivity Analysis: Multivariable-adjusted 3-year Estimated Changes^a^ in Rescaled Short Physical Performance Battery (SPPB)^b^ Scores and Grip Strength^c^ by Randomized Intervention Assignment, The Aging and Cognitive Health Evaluation in Elders (ACHIEVE) study, N = 891, 2018−22. (PDF)

S2 Fig Sensitivity Analysis of the Complier-average Causal Effect: Per-protocol Sensitivity Analysis: Multivariable-adjusted 3-year Estimated Changes^a^ in Rescaled Short Physical Performance Battery (SPPB)^b^ Scores and Grip Strength^c^ by Randomized Intervention Assignment and Recruitment Sourced, The Aging and Cognitive Health Evaluation in Elders (ACHIEVE) study, N = 891, 2018−22. (PDF)
