# Supplementary material for: Effects of hearing intervention on physical function: A secondary analysis of the ACHIEVE study
Source: PLoS One. 2026 Apr 29;21(4):e0347500. doi: 10.1371/journal.pone.0347500 (PMC13127907; doi:10.1371/journal.pone.0347500)
Supplement: S2 File — (PDF) [file pone.0347500.s002.pdf]

**Effects of Hearing Intervention on Physical Function: A Secondary Analysis of the ACHIEVE Study**
**Deal JA et al.**
**S2. CONSORT Checklist**

| Table. CONSORT 2010 Checklist of Information to Include When Reporting a Randomized Trial | | | |
|---|---|---|---|
| **Section and Topic** | **Item No.** | **Checklist items** | **Reported on Page No.** |
| **Title and abstract** | 1a | Identification as a randomized trial in the title | 1 |
| | 1b | Structured summary of trial design, methods, results, and conclusions (for specific guidance see CONSORT for abstracts) | 2 |
| **Introduction** | 2a | Scientific background and explanation of rationale | 3 |
| Background and objectives | 2b | Specific objectives or hypotheses | 3 |
| **Methods** Trial design | 3a | Description of trial design (such as parallel, factorial) including allocation ratio | 3-4 |
| | 3b | Important changes to methods after trial commencement (such as eligibility criteria), with reasons | 4-5 |
| Participants | 4a | Eligibility criteria for participants | 4 |
| | 4b | Settings and locations where the data were collected | 4 |
| Interventions | 5 | The interventions for each group with sufficient details to allow replication, including how and when they were actually administered | 5 |
| Outcomes | 6a | Completely defined prespecified primary and secondary outcome measures, including how and when they were assessed | 6-8 |
| | 6b | Any changes to trial outcomes after the trial commenced, with reasons | n/a |
| Sample size | 7a | How sample size was determined | 5,7 |
| | 7b | When applicable, explanation of any interim analyses and stopping guidelines | 7 |
| Randomization Sequence generation | 8a | Method used to generate the random allocation sequence | 8 |
| | 8b | Type of randomization; details of any restriction (such as blocking and block size) | 8 |
| Allocation concealment mechanism | 9 | Mechanism used to implement the random allocation sequence(such as sequentially numbered containers), describing any steps taken to conceal the sequence until interventions were assigned | 8 |
| Implementation | 10 | Who generated the random allocation sequence, who enrolled participants, and who assigned participants to interventions | 8 |
| Masking | 11a | If done, who was blinded after assignment to interventions (for example, participants, care providers, those assessing outcomes) and how | 8 |
| | 11b | If relevant, description of the similarity of interventions | n/a |
| Statistical methods | 12a | Statistical methods used to compare groups for primary and secondary outcomes | 8-11 |
| | 12b | Methods for additional analyses, such as subgroup analyses and adjusted analyses | 8-11 |
| **Results** Participant flow (a diagram is strongly recommended) | 13a | For each group, the numbers of participants who were randomly assigned, received intended treatment, and were analyzed for the primary outcome | 11, Fig 1 |
| | 13b | For each group, losses and exclusions after randomization, together with reasons | 4, Fig 1 |
| Recruitment | 14a | Dates defining the periods of recruitment and follow-up | 4-5 |
| | 14b | Why the trial ended or was stopped | 4-5 |
| Baseline data | 15 | A table showing baseline demographic and clinical characteristics for each group | 12-13 |
| Numbers analyzed | 16 | For each group, number of participants (denominator) included in each analysis and whether the analysis was by original assigned groups | Fig 1 |
| Outcomes and estimation | 17a | For each primary and secondary outcome, results for each group, and the estimated effect size and its precision (such as 95% confidence interval) | 14-17 |
| | 17b | For binary outcomes, presentation of both absolute and relative effect sizes is recommended | n/a |
| Ancillary analyses | 18 | Results of any other analyses performed, including subgroup analyses and adjusted analyses, distinguishing prespecified from exploratory | 17 |

**Effects of Hearing Intervention on Physical Function: A Secondary Analysis of the ACHIEVE Study**
**Deal JA et al.**
**S2. CONSORT Checklist**

| | | | |
|---|---|---|---|
| Harms | 19 | All important harms or unintended effects in each group (for specific guidance see CONSORT for harms) | 11 |
| **Comment**<br>Limitations | 20 | Trial limitations, addressing sources of potential bias, imprecision, and, if relevant, multiplicity of analyses | 19 |
| Generalizability | 21 | Generalizability (external validity, applicability) of the trial findings | 19 |
| Interpretation | 22 | Interpretation consistent with results, balancing benefits and harms, and considering other relevant evidence | 17-20 |
| **Other information**<br>Registration | 23 | Registration number and name of trial registry | 2,3 |
| Protocol | 24 | Where the full trial protocol can be accessed, if available | 4, Suppleme-ntal file S2 |
| Funding | 25 | Sources of funding and other support (such as supply of drugs), role of funders | Published with the manuscript |

Source: Hopewell S, Chan A, Collins GS, et al. CONSORT 2025 Statement: Updated Guideline for Reporting Randomized Trials. *JAMA.* 2025;333(22):1998–2005. doi:10.1001/jama.2025.4347.
