# Supplementary material for: Effects of hearing intervention on physical function: A secondary analysis of the ACHIEVE study
Source: PLoS One. 2026 Apr 29;21(4):e0347500. doi: 10.1371/journal.pone.0347500 (PMC13127907; doi:10.1371/journal.pone.0347500)
Supplement: S3 File — Supplemental Methods. (PDF) [file pone.0347500.s003.pdf]

Effects of Hearing Intervention on Physical Function: A Secondary Analysis of the ACHIEVE Study
Deal JA et al.
S1 Appendix. Supplemental Methods

Recruitment Details

Recruitment details have been previously described.[1] The ACHIEVE study is based within the Atherosclerosis Risk in Communities (ARIC) study, an ongoing longitudinal study in four field sites (Forsyth County, North Carolina, USA; Jackson, Mississippi, USA; Minneapolis, Minnesota, USA; and Washington County, Maryland, USA).[2] ACHIEVE participants (N=977) were recruited from the four ARIC sites between 2018-2019; 238 participants were from ARIC-NCS, and 739 participants were de novo volunteers from the community. A soft-launch of recruitment efforts began at a limited number of study sites in November 2017 (Washington County, Minneapolis, Forsyth County) and full-scale efforts at all sites in January (Washington County, Minneapolis, Forsyth County) or February (Mississippi) 2018. The original target sample size (N=850) was achieved in July 2019. After interim Data Safety and Monitoring Board (DSMB) analysis of drop-in, drop-out and missing data, and prior to recruitment closure, with National Institute of Aging approval, investigators were authorized to extend the recruitment window, which closed on October 27, 2019 with a final sample of N=977. The trial concluded as scheduled at the end of 3 years. The Institutional Review Boards (IRBs) at each collaborating site, including the 4 field centers and the coordinating center (University of Mississippi Medical Center IRB, University of Minnesota IRB, Wake Forest University IRB, Johns Hopkins Bloomberg School of Public Health IRB, University of North Carolina IRB) approved the trial, and participants provided written informed consent. An independent data and safety monitoring board oversaw study progress, adverse events, and changes to the study protocol and statistical analysis plan.[1]

Rescaling Short Physical Performance Battery (SPPB) Scores

Given the ceiling effects in high functioning individuals in our study population, we rescaled SPPB scores according to published guidelines developed in the Health Aging and Body Composition study.[3] The table below provides a detailed illustration of the measurement of SPPB components and the algorithm adopted to derive the rescaled score.

SPPB component	Measurement	Scoring	Conversion
Chair Stand	With arms folded across, participants were asked to stand up from an armless chair five times as quickly as possible. Time spent completing the task was recorded.	Participants were assigned a score of zero if they refused/were unable to perform the task or failed to complete all five stands.	For each participant, we calculated the rate of chair stand equal to five divided by the total time spent. Rates were right truncated at 1 chair stand per second (n=3) as the final chair stand component score.
Balance	Participants were first asked to balance in a semi-tandem position for 10 seconds. Those who completed the task were asked to balance in a full tandem position (with two attempts) for up to 10 seconds. Those who were unable to hold the semi-tandem task for the full 10 seconds were asked to complete a side-by-side stand for up to 10 seconds.	Participants who completed the semi-tandem stand scored 20+X seconds (X is the longer time recorded in the two attempts of the tandem position, $X \leq 10$). Participants who did not complete the semi-tandem stand scored Y+Z seconds (Y and Z are the actual time recorded holding semi-tandem and side-by-side stand, $Y<10$ and $Z \leq 10$)	Participants who completed the semi-tandem stand were assumed to be able to complete the easier side-by-side task and were scored as if it was held for the full time (10 seconds). Individual scores were divided by 30 to be converted to a final balance component score with the range of 0 to 1.
4-meter walk	Participants were asked to complete two 4-meter walking trials at their usual walking speed without walking aids (if possible). Time spent on each trial was recorded separately.	An average walking speed of the two trials was calculated using the formula: 8 meters / (time spent on trial 1 + time spent on trial 2, in seconds).	The final 4-meter walk component score was derived by dividing the average walking speed of the two trails by 2 m/s. This score took a range between 0 and 1.

Multiple Imputation Methods

We replicated our primary analysis using multiple imputation by chained equations[4] to impute missing covariate and outcome data. The imputation model included all covariates from the fully adjusted model (age, sex, race, field site, education, recruitment source, BMI, and hearing loss severity), as well as auxiliary variables[5,6] from baseline that measured arthritis (present versus not), living arrangements (living alone versus not), cigarette use (ever versus never), and leisure-time and sport-related physical activity as quantified by the Baecke Physical Activity Questionnaire[5] (summary score ranging from 1 to 5, with 0.25 increments). We specified an interaction between each measure in the imputation model and time, treatment assignment, and recruitment source. We also included three-way interaction terms between time, treatment assignment, and measures of physical function (separately for three component scores) and physical activity. We generated 100 imputed datasets based on a two-stage analysis suggesting that precision would be acceptable if a minimum of 97 datasets were analyzed.[8]

Only pre-death measures of SPPB components and grip strength were analyzed. We accomplished this by constructing three separate imputation sub-models. The first sub-model included all analytic and auxiliary variables from the baseline and imputed missing measures among all participants (N=977). The second sub-model included all analytic and auxiliary variables from the baseline and 1-year follow-up and imputed missing measures at the 1-year follow-up among living participants (N=973). The third sub-model included all analytic and auxiliary variables from the baseline, 1-year follow-up, and 3-year follow-up and imputed missing measures among participants alive at the 3-year follow-up (N=943). We integrated the imputed datasets into a composite dataset in which concurrent and past variables, but not future variables, informed the values generated for missing measures of SPPB components and grip strength among living participants. Parameter estimates from models fit to imputed data were combined according to Rubin's rules.[9]

Effect of Treatment Adherence

To evaluate the effect of treatment adherence, we initially performed a per-protocol analysis of imputed data. The analysis included participants who completed the intervention, had no major protocol deviations, never wore hearing aids if they were assigned to the control, and never discontinued hearing aid use if they were assigned to the intervention (Total N=864, Intervention N=478, Control N=386).

To reduce selection bias present in per-protocol estimates [8, 9], we further estimated the complier average causal effect (CACE) in the entire sample (N=977, Intervention N=490, Control N=487) using inverse probability weights.[12-15] First, we fit a logistic regression model among participants who were assigned to receive hearing intervention (N=490), the model included treatment compliance (0 if dropped out of the intervention and 1 if completed the intervention) as the dependent variable and a set of covariates (age, sex, field site, education, recruitment source, and presence of depressive symptoms as determined by the Center for Epidemiologic Studies-Depression (CES-D) scale) as predictors. The Receiver Operating Characteristic Curve (ROC) was used to evaluate model performance, and an Area Under the Curve (AUC) value of 0.73 was calculated, indicating an acceptable level of prediction power. Second, we generated the CACE weights by estimating the inverse probability of compliance in all study participants based on the parameter estimates obtained from the logistic regression model. We then fit a weighted, fully adjusted model (with all covariates specified as in our primary analyses) to the imputed data to estimate the effect of treatment adherence conditional on the assumption of principal ignorability.[16]

References

1. Reed NS, Gravens-Mueller L, Huang AR, et al. for the ACHIEVE Collaborative Research Group. Recruitment and baseline data of the Aging and Cognitive Health Evaluation in Elders (ACHIEVE) study: A randomized trial of a hearing loss intervention for reducing cognitive decline. *Alzheimers Dement* (N Y). 2024 Feb 14;10(1):e12453. doi: 10.1002/trc2.12453. PMID: 38356470; PMCID: PMC10865776.
2. Wright JD, Folsom AR, Coresh J, et al. The ARIC (Atherosclerosis Risk In Communities) Study: JACC Focus Seminar 3/8. *J Am Coll Cardiol*. 2021 Jun 15;77(23):2939-2959. doi: 10.1016/j.jacc.2021.04.035. PMID: 34112321; PMCID: PMC8667593.
3. Simonsick EM, Newman AB, Nevitt MC, et al. Measuring higher level physical function in well-functioning older adults: Expanding familiar approaches in the Health ABC study. *J Gerontol A Biol Sci Med Sci*. 2001;56(10):M644-9.
4. Van Buuren S. Multiple imputation of discrete and continuous data by fully conditional specification. *Stat Methods Med Res.* 2007;16(3):219-242. doi: 10.1177/0962280206074463
5. Collins LM, Schafer JL, Kam CM. A comparison of inclusive and restrictive strategies in modern missing data procedures. *Psychol Methods*. 2001;6(4):330-351. doi:10.1037/1082-989X.6.4.330.
6. Mainzer RM, Nguyen CD, Carlin JB, Moreno-Betancur M, White IR, Lee KJ. A comparison of strategies for selecting auxiliary variables for multiple imputation. *Biom J*. 2024;66(1):2200291. doi:10.1002/bimj.202200291.
7. Baecke J, Burema J, Frijters J. A short questionnaire for the measurement of habitual physical activity in epidemiological studies. *Am J Clin Nutr.* 1982;36(5):936-942. doi:10.1093/ajcn/36.5.936.
8. von Hippel P. How many imputations do you need? A two-stage calculation using a quadratic rule. *Sociol Methods Res*. 2020;49(3):699-718. doi: 10.1177/0049124117747303.
9. Rubin DB. Multiple Imputation for Nonresponse in Surveys. John Wiley & Sons; 2004.

10. Little R, Rubin D. Causal effects in clinical and epidemiological studies via potential outcomes: Concepts and analytical approaches. *Annu Rev Public Health*. 2000;21:121-145. doi: 10.1146/annurev.publhealth.21.1.121.

11. Shrier I, Steele R, Verhagen E, Herbert R, Riddell C, Kaufman J. Beyond intention to treat: What is the right question? *Clin Trials*. 2014;11(1):28-37. doi: 10.1177/1740774513504151.

12. Little RJ, Rubin DB. Causal Effects in Clinical and Epidemiological Studies Via Potential Outcomes: Concepts and Analytical Approaches. *Annu Rev Public Health*. 2000;21(Volume 21, 2000):121-145. doi:10.1146/annurev.publhealth.21.1.121

13. Shrier I, Steele RJ, Verhagen E, Herbert R, Riddell CA, Kaufman JS. Beyond intention to treat: what is the right question? *Clin Trials*. 2014 Feb;11(1):28-37. doi: 10.1177/1740774513504151. Epub 2013 Oct 3. PMID: 24096636.

14. Hernán M, Robins J. Per-protocol analyses of pragmatic trials. *N Engl J Med*. 2017;377(14):1391-1398. doi: 10.1056/NEJMsm1605385.

15. Jo B, Stuart E. On the use of propensity scores in principal causal effect estimation. *Stat Med*. 2009;28(23):2857-2875. doi: 10.1002/sim.3669.

16. Herbert R, Kasza J, Bø K. Analysis of randomised trials with long-term follow-up. *BMC Med Res Methodol.* 2018;18(1):48. doi: 10.1186/s12874-018-0499-5.
