# Supplementary material for: Effects of hearing intervention on physical function: A secondary analysis of the ACHIEVE study
Source: PLoS One. 2026 Apr 29;21(4):e0347500. doi: 10.1371/journal.pone.0347500 (PMC13127907; doi:10.1371/journal.pone.0347500)
Supplement: S1 Table — (PDF) [file pone.0347500.s004.pdf]

Effects of Hearing Intervention on Physical Function: A Secondary Analysis of the ACHIEVE Study.
Deal JA et al. Supplemental Tables.

S4 Supplemental Table 1. Distributions of Baseline and Follow-up of Total Short Physical Performance Battery (SPPB) and Grip Strength Scores, by Randomized Intervention Assignment, Recruitment Source, and Sex (Grip Strength Only), The Aging and Cognitive Health Evaluation in Elders (ACHIEVE) study, N=956, 2018-22

	**Mean (SD)**								
	**Overall**	**Total Cohort (n=956)**		**ARIC (n=226)**			**De novo (n=730)**		
		**Control**	**Intervention**	**Total**	**Control**	**Intervention**	**Total**	**Control**	**Intervention**
	N=956	N=477	N=479	N=226	N=112	N=114	N=730	N=365	N=365
**Original SPPB score**									
Baseline (N=944)	10.0 (2.0)	9.9 (2.1)	10.0 (2.0)	9.4 (2.4)	9.2 (2.5)	9.5 (2.4)	10.1 (1.8)	10.2 (1.9)	10.1 (1.8)
Year 1 (N=580)	9.9 (2.1)	9.7 (2.2)	10.0 (2.1)	9.3 (2.4)	9.2 (2.3)	9.4 (2.4)	10.1 (1.9)	10.0 (2.1)	10.3 (1.8)
Year 3 (N=821)	9.1 (2.6)	9.0 (2.6)	9.3 (2.6)	8.5 (3.0)	8.2 (3.1)	8.8 (2.8)	9.3 (2.5)	9.3 (2.4)	9.4 (2.5)
**Rescaled SPPB score**									
Baseline (N=956)	1.8 (0.3)	1.8 (0.3)	1.8 (0.3)	1.7 (0.4)	1.7 (0.4)	1.7 (0.4)	1.8 (0.3)	1.8 (0.3)	1.8 (0.3)
Year 1 (N=585)	1.8 (0.3)	1.8 (0.4)	1.8 (0.3)	1.7 (0.4)	1.7 (0.4)	1.7 (0.4)	1.8 (0.3)	1.8 (0.3)	1.9 (0.3)
Year 3 (N=837)	1.7 (0.5)	1.6 (0.5)	1.7 (0.5)	1.6 (0.5)	1.5 (0.5)	1.6 (0.5)	1.7 (0.4)	1.7 (0.4)	1.7 (0.5)
**Grip Strength, kg**									
Overall									
Baseline (N=956)	28.0 (9.7)	27.8 (9.6)	28.2 (9.8)	26.9 (8.8)	26.6 (8.2)	27.3 (9.4)	28.3 (9.9)	28.1 (9.9)	28.5 (9.9)
Year 1 (N=585)	26.8 (9.6)	26.5 (9.2)	27.1 (9.9)	26.5 (8.9)	26.1 (8.6)	27.0 (9.3)	26.9 (9.9)	26.7 (9.6)	27.1 (10.2)
Year 3 (N=829)	25.4 (9.2)	25.2 (8.9)	25.7 (9.5)	25.3 (8.5)	24.7 (8.2)	25.9 (8.9)	25.5 (9.4)	25.3 (9.2)	25.7 (9.6)
Males									
Baseline (N=447)	35.5 (8.1)	35.1 (7.9)	35.9 (8.3)	34.9 (7.2)	33.4 (7.2)	36.4 (7.1)	35.6 (8.3)	35.5 (8.1)	35.7 (8.5)
Year 1 (N=257)	34.4 (8.3)	33.9 (7.6)	34.9 (8.9)	34.4 (7.8)	33.3 (7.7)	35.6 (7.8)	34.4 (8.5)	34.1 (7.6)	34.7 (9.3)
Year 3 (N=379)	32.3 (8.3)	31.8 (7.8)	32.8 (8.7)	33.2 (7.3)	31.8 (7.9)	34.6 (6.4)	32.1 (8.4)	31.8 (7.8)	32.4 (9.0)
Females									
Baseline (N=509)	21.4 (5.1)	21.2 (5.1)	21.6 (5.2)	21.9 (5.4)	22.3 (5.4)	21.6 (5.4)	21.2 (5.0)	20.8 (5.0)	21.5 (5.1)
Year 1 (N=328)	20.8 (5.3)	20.5 (5.2)	21.1 (5.4)	21.7 (5.5)	21.6 (5.6)	21.8 (5.4)	20.2 (5.1)	19.8 (4.8)	20.6 (5.3)
Year 3 (N=450)	19.7 (5.2)	19.5 (5.2)	19.9 (5.2)	21.1 (5.8)	21.2 (5.6)	21.1 (6.0)	19.2 (4.8)	18.9 (4.9)	19.4 (4.8)

Abbreviations: ARIC, The Atherosclerosis Risk in Communities Study; SD, standard deviation; SPPB, Short Physical Performance Battery
