# Supplementary material for: Effects of hearing intervention on physical function: A secondary analysis of the ACHIEVE study
Source: PLoS One. 2026 Apr 29;21(4):e0347500. doi: 10.1371/journal.pone.0347500 (PMC13127907; doi:10.1371/journal.pone.0347500)
Supplement: S2 Table — (PDF) [file pone.0347500.s005.pdf]

Effects of Hearing Intervention on Physical Function: A Secondary Analysis of the ACHIEVE Study.
Deal JA et al. Supplemental Tables.

S5. Supplemental Table 2. Distributions of Baseline and Follow-up Short Physical Performance Battery (SPPB) Component Scores (Original and Transformed), by Randomized Intervention Assignment and Recruitment Source, The Aging and Cognitive Health Evaluation in Elders (ACHIEVE) study, N=956, 2018-22

	**Mean (SD)**								
	**Overall**	**Total Cohort (N=956)**		**ARIC (n=226)**			**De novo (n=730)**		
		Control	Intervention	Total	Control	Intervention	Total	Control	Intervention
	N=956	N=477	N=479	N=226	N=112	N=114	N=730	N=365	n=365
**Chair stands**									
Time to complete (s)									
Baseline (N=870)	13.2 (4.0)	13.2 (3.7)	13.3 (4.2)	14.0 (4.2)	13.8 (3.4)	14.2 (4.8)	13.0 (3.9)	13.0 (3.8)	13.0 (4.0)
Year 1 (N=536)	13.3 (3.9)	13.4 (4.2)	13.1 (3.7)	13.7 (4.0)	13.8 (3.9)	13.5 (4.1)	13.1 (3.9)	13.2 (4.3)	13.0 (3.4)
Year 3 (N=706)	14.4 (4.2)	14.6 (4.4)	14.2 (3.9)	15.0 (4.3)	15.4 (4.6)	14.7 (4.1)	14.2 (4.1)	14.4 (4.3)	14.0 (3.9)
Number/s[a]									
Baseline (N=956)	0.4 (0.2)	0.4 (0.2)	0.4 (0.2)	0.3 (0.2)	0.3 (0.2)	0.4 (0.2)	0.4 (0.2)	0.4 (0.2)	0.4 (0.2)
Year 1 (N=588)	0.4 (0.2)	0.4 (0.2)	0.4 (0.2)	0.3 (0.2)	0.3 (0.2)	0.4 (0.2)	0.4 (0.2)	0.4 (0.2)	0.4 (0.1)
Year 3 (N=837)	0.3 (0.2)	0.3 (0.2)	0.3 (0.2)	0.3 (0.2)	0.3 (0.2)	0.3 (0.2)	0.3 (0.2)	0.3 (0.2)	0.3 (0.2)
Rescaled score[b]									
Baseline (N=956)	0.4 (0.2)	0.4 (0.2)	0.4 (0.2)	0.3 (0.2)	0.3 (0.2)	0.4 (0.2)	0.4 (0.2)	0.4 (0.2)	0.4 (0.2)
Year 1 (N=588)	0.4 (0.2)	0.4 (0.2)	0.4 (0.2)	0.3 (0.2)	0.3 (0.2)	0.4 (0.2)	0.4 (0.2)	0.4 (0.2)	0.4 (0.1)
Year 3 (N=837)	0.3 (0.2)	0.3 (0.2)	0.3 (0.2)	0.3 (0.2)	0.3 (0.2)	0.3 (0.2)	0.3 (0.2)	0.3 (0.2)	0.3 (0.2)
**4-meter walk**									
Time to complete (s)									
Baseline (N=956)	4.3 (1.2)	4.4 (1.3)	4.3 (1.2)	4.8 (1.7)	4.8 (1.9)	4.8 (1.6)	4.2 (1.0)	4.2 (1.1)	4.1 (0.9)
Year 1 (N=588)	4.4 (1.3)	4.4 (1.3)	4.4 (1.3)	4.8 (1.6)	4.7 (1.6)	4.8 (1.6)	4.2 (1.1)	4.3 (1.1)	4.2 (1.2)
Year 3 (N=817)	4.6 (1.4)	4.6 (1.4)	4.6 (1.5)	5.0 (1.8)	5.0 (1.8)	5.0 (1.7)	4.5 (1.3)	4.5 (1.2)	4.5 (1.4)
Speed (m/s)[a]									
Baseline (N=956)	1.0 (0.2)	1.0 (0.2)	1.0 (0.2)	0.9 (0.2)	0.9 (0.2)	0.9 (0.2)	1.0 (0.2)	1.0 (0.2)	1.0 (0.2)
Year 1 (N=589)	1.0 (0.2)	1.0 (0.2)	1.0 (0.2)	0.9 (0.2)	0.9 (0.2)	0.9 (0.2)	1.0 (0.2)	1.0 (0.2)	1.0 (0.2)
Year 3 (N=837)	0.9 (0.3)	0.9 (0.2)	0.9 (0.3)	0.8 (0.3)	0.8 (0.3)	0.9 (0.3)	0.9 (0.2)	0.9 (0.2)	0.9 (0.3)
Rescaled score[b]									
Baseline (N=956)	0.5 (0.1)	0.5 (0.1)	0.5 (0.1)	0.5 (0.1)	0.5 (0.1)	0.5 (0.1)	0.5 (0.1)	0.5 (0.1)	0.5 (0.1)
Year 1 (N=589)	0.5 (0.1)	0.5 (0.1)	0.5 (0.1)	0.5 (0.1)	0.5 (0.1)	0.5 (0.1)	0.5 (0.1)	0.5 (0.1)	0.5 (0.1)
Year 3 (N=837)	0.5 (0.1)	0.4 (0.1)	0.5 (0.1)	0.4 (0.1)	0.4 (0.1)	0.4 (0.1)	0.5 (0.1)	0.5 (0.1)	0.5 (0.1)
**Standing Balance**									

Effects of Hearing Intervention on Physical Function: A Secondary Analysis of the ACHIEVE Study.
Deal JA et al. Supplemental Tables.

Time (s)									
Baseline (N=956)	28.2 (4.7)	28.2 (4.8)	28.2 (4.7)	27.1 (6.4)	27.0 (6.3)	27.1 (6.5)	28.6 (4.0)	28.6 (4.2)	28.6 (3.9)
Year 1 (N=585)	28.0 (4.8)	27.7 (5.0)	28.3 (4.7)	27.2 (5.6)	26.9 (5.5)	27.5 (5.8)	28.4 (4.3)	28.1 (4.6)	28.7 (4.0)
Year 3 (N=837)	26.6 (7.3)	26.5 (7.4)	26.8 (7.2)	25.7 (7.9)	24.9 (8.7)	26.6 (6.9)	26.9 (7.1)	26.9 (6.9)	26.8 (7.3)
Ability to hold, N(%)[a,c]									
Baseline									
Yes	764 (79.9)	380 (79.7)	384 (80.2)	161 (71.2)	77 (68.8)	84 (73.7)	603 (82.6)	303 (83.0)	300 (82.2)
No	192 (20.1)	97 (20.3)	95 (19.8)	65 (28.8)	35 (31.2)	30 (26.3)	127 (17.4)	62 (17.0)	65 (17.8)
Year 1									
Yes	450 (47.1)	218 (45.7)	232 (48.4)	132 (58.4)	64 (57.1)	68 (59.6)	318 (43.6)	154 (42.2)	164 (44.9)
No	135 (14.1)	75 (15.7)	60 (12.5)	68 (30.1)	37 (33.0)	31 (27.2)	67 (9.2)	38 (10.4)	29 (7.9)
Missing	371 (38.8)	184 (38.6)	187 (39.0)	26 (11.5)	11 (9.8)	15 (13.2)	345 (47.3)	173 (47.4)	172 (47.1)
Year 3									
Yes	591 (61.8)	289 (60.6)	302 (63.0)	120 (53.1)	58 (51.8)	62 (54.4)	471 (64.5)	231 (63.3)	240 (65.8)
No	246 (25.7)	128 (26.8)	118 (24.6)	67 (29.6)	39 (34.8)	28 (24.6)	179 (24.5)	89 (24.4)	90 (24.7)
Missing	119 (12.4)	60 (12.6)	59 (12.3)	39 (17.3)	15 (13.4)	24 (21.1)	80 (11.0)	45 (12.3)	35 (9.6)
Rescaled score[b]									
Baseline (N=956)	0.9 (0.2)	0.9 (0.2)	0.9 (0.2)	0.9 (0.2)	0.9 (0.2)	0.9 (0.2)	1.0 (0.1)	1.0 (0.1)	1.0 (0.1)
Year 1 (N=585)	0.9 (0.2)	0.9 (0.2)	0.9 (0.2)	0.9 (0.2)	0.9 (0.2)	0.9 (0.2)	0.9 (0.1)	0.9 (0.2)	1.0 (0.1)
Year 3 (N=837)	0.9 (0.2)	0.9 (0.2)	0.9 (0.2)	0.9 (0.3)	0.8 (0.3)	0.9 (0.2)	0.9 (0.2)	0.9 (0.2)	0.9 (0.2)

Abbreviations: ARIC, The Atherosclerosis Risk in Communities Study; SD, standard deviation

[a] Transformation used for primary analysis

[b] Scores rescaled for high-functioning populations according to published guidelines developed in the Health Aging and Body Composition (Health ABC) study (Simonsick EM, Newman AB, Nevitt MC, et al. Measuring higher level physical function in well-functioning older adults: Expanding familiar approaches in the Health ABC study. *J Gerontol A Biol Sci Med Sci*. 2001;56(10):M644-9).

[c] Standing balance was modeled as a binary variable (no vs. yes [reference]), as the ability to hold all three positions (side-by-side, semi-tandem, full-tandem) for the time.
