# Supplementary material for: Effects of hearing intervention on physical function: A secondary analysis of the ACHIEVE study
Source: PLoS One. 2026 Apr 29;21(4):e0347500. doi: 10.1371/journal.pone.0347500 (PMC13127907; doi:10.1371/journal.pone.0347500)
Supplement: S6 Table — (PDF) [file pone.0347500.s009.pdf]

Effects of Hearing Intervention on Physical Function: A Secondary Analysis of the ACHIEVE Study
Deal JA et al. Supplemental Tables.

S9. Supplemental Table 6. Sensitivity Analysis with Multiple Imputation to Impute Missing Data: Multivariable-adjusted 3-Year Estimated Changes in Components of the Short Physical Performance Battery (Chair Stands, 4-meter Walk, Balance) by Randomized Intervention Assignment and Recruitment Source, The Aging and Cognitive Health Evaluation in Elders (ACHIEVE) study, N=977, 2018-22

	**Total Cohort[a]**	**ARIC[b]**	**De novo[b]**
	**Estimate (95% CI)**	**Estimate (95% CI)**	**Estimate (95% CI)**
**Chair stands[c]**			
Intervention	-0.82 (-0.95, -0.69)	-0.69 (-0.88, -0.50)	-0.83 (-0.96, -0.69)
Control	-0.84 (-0.98, -0.71)	-0.77 (-0.96, -0.59)	-0.83 (-0.97, -0.70)
Treatment effect (Rate difference)[d]	0.03 (-0.07, 0.12)	0.08 (-0.12, 0.28)	0.01 (-0.11, 0.12)
**4-meter walk[c]**			
Intervention	-0.33 (-0.49, -0.16)	-0.09 (-0.33, 0.16)	-0.35 (-0.53, -0.18)
Control	-0.26 (-0.43, -0.09)	-0.19 (-0.43, 0.04)	-0.24 (-0.41, -0.06)
Treatment effect (Rate difference)[d]	-0.06 (-0.19, 0.06)	0.10 (-0.15, 0.36)	-0.12 (-0.26, 0.02)
**Standing balance[e]**			
Intervention (Log odds)	0.55 (0.04, 1.06)	0.32 (-0.35, 0.98)	0.57 (0.04, 1.09)
Control (Log odds)	0.63 (0.12, 1.14)	0.49 (-0.18, 1.15)	0.62 (0.08, 1.15)
Treatment effect (Log odds difference)[d]	-0.08 (-0.45, 0.29)	-0.17 (-0.82, 0.48)	-0.05 (-0.51, 0.41)
Treatment effect (Odds ratio)[d]	0.92 (0.64, 1.34)	0.84 (0.44, 1.61)	0.95 (0.60, 1.50)

Abbreviations: ARIC, The Atherosclerosis Risk in Communities Study; CI, confidence interval

[a] Estimates in the total cohort for chair stands and 4-meter walk were obtained using linear mixed effects models with random intercepts, random slopes, and unstructured covariance. Estimates in the total cohort for standing balance were obtained using Generalized Estimating Equations with unstructured correlation matrix and robust standard errors. Models included treatment, time since baseline, and an interaction term between time and treatment. Models adjusted for age, sex, race, field site, education, recruitment source, body mass index, and pure-tone average.
[b] Estimates by recruitment source for chair stands and 4-meter walk were obtained using linear mixed effects models with random intercepts, random slopes, and unstructured covariance. Estimates by recruitment source for standing balance were obtained using Generalized Estimating Equations with unstructured correlation matrix and robust standard errors. Models included treatment, recruitment source, time since baseline, an interaction term between treatment and recruitment source, an interaction term between time and treatment, an interaction term between time and recruitment source, and a three-way interaction term between time, treatment and recruitment source. Models adjusted for age, sex, race, field site, education, body mass index, and pure-tone average.
[c] Chair stand rates (number of chair stands/second) and 4-meter walk speeds (m/s) were standardized for analysis by subtracting baseline mean and then dividing by baseline standard deviation.
[d] For chair stands and 4-m walk, the treatment effect is the estimated difference in 3-year rates of change in the outcome comparing intervention to control. For balance, treatment effects are presented as the difference in the 3-year log odds and odds ratio comparing intervention to control.
[e] Standing balance was modeled as a binary variable (no vs. yes [reference]), as the ability to hold all three positions (side-by-side, semi-tandem, full-tandem) for the time.
