# Supplementary material for: Effects of hearing intervention on physical function: A secondary analysis of the ACHIEVE study
Source: PLoS One. 2026 Apr 29;21(4):e0347500. doi: 10.1371/journal.pone.0347500 (PMC13127907; doi:10.1371/journal.pone.0347500)
Supplement: S2 Fig — (PDF) [file pone.0347500.s014.pdf]

Effects of Hearing Intervention on Physical Function: A Secondary Analysis of the ACHIEVE Study
Deal JA et al. Supplemental Figures

S14. Supplemental Figure 2. Sensitivity Analysis of the Complier-average Causal Effect: Per-protocol Sensitivity Analysis: Multivariable-adjusted 3-year Estimated Changes[a] in Rescaled Short Physical Performance Battery (SPPB)[b] Scores and Grip Strength[c] by Randomized Intervention Assignment and Recruitment Source[d], The Aging and Cognitive Health Evaluation in Elders (ACHIEVE) study, N=891, 2018-22

A. Total Cohort

	3-Year Change β (95% CI)	Treatment Effect[e] β (95% CI)
Rescaled SPPB score (n=977)		
Control	-0.170 (-0.538, 0.198)	-0.001 (-0.114, 0.112)
Intervention	-0.171 (-0.551, 0.209)	
Mean Grip Strength (n=977)		
Control	-0.208 (-0.672, 0.256)	0.016 (-0.053, 0.085)
Intervention	-0.192 (-0.656, 0.272)	

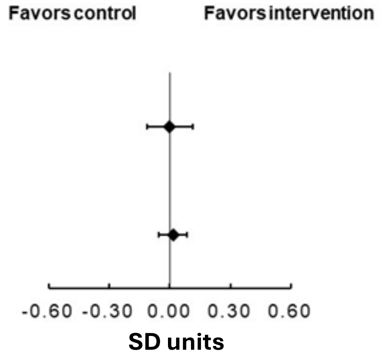

B. ARIC

	3-Year Change β (95% CI)	Treatment Effect[e] β (95% CI)
Rescaled SPPB score (n=238)		
Control	0.442 (-0.358, 1.242)	0.243 (-0.000, 0.487)
Intervention	0.685 (-0.106, 1.477)	
Mean Grip Strength (n=238)		
Control	-0.377 (-0.783, 0.029)	0.019 (-0.099, 0.138)
Intervention	-0.357 (-0.754, 0.039)	

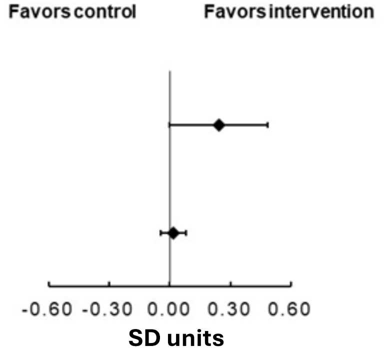

C. De novo

	3-Year Change β (95% CI)	Treatment Effect[e] β (95% CI)
Rescaled SPPB score (n=739)		
Control	-0.302 (-0.699, 0.095)	-0.069 (-0.195, 0.056)
Intervention	-0.371 (-0.780, 0.038)	
Mean Grip Strength (n=739)		
Control	-0.165 (-0.717, 0.386)	0.016 (-0.066, 0.099)
Intervention	-0.149 (-0.704, 0.406)	

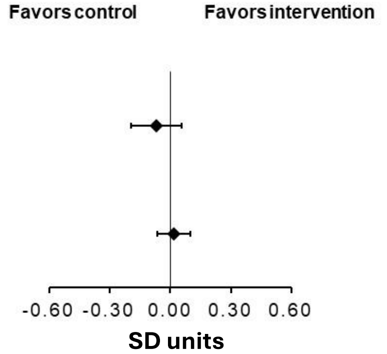

Abbreviations: ARIC, The Atherosclerosis Risk in Communities Study; CI, confidence interval; SPPB, Short Physical Performance Battery

[a] Estimates in the total cohort were obtained using linear mixed effects models with random intercepts, random slopes, and unstructured covariance. Models included treatment, time since baseline, and an interaction term between time and treatment. Models adjusted for age, sex, race, field site, education, recruitment source, body mass index, and pure-tone average.

[b] The rescaled SPPB score includes chair stand rate (chair stands/second), standing balance (total time in seconds participants held in side-by-side, semi-tandem, full-tandem) and 4-meter walking speed (meter/second). For each component, participants' performance was divided by maximum performance (1 chair stand/second; 30 seconds; 2 meters/second) and was thus converted to a ratio ranging from 0-1. Total rescaled SPPB scores range from 0-3; higher score indicates better performance. The rescaled SPPB score was standardized for analysis by subtracting baseline mean and then dividing by baseline standard deviation.
[c] Grip strength was assessed by dynamometer and the strength in kilograms obtained from two test trials were averaged. Grip strength was standardized by subtracting baseline mean and then dividing by baseline standard deviation.
[d] Estimates by recruitment source were obtained using linear mixed effects models with random intercepts, random slopes, and unstructured covariance. Models included treatment, recruitment source, time since baseline, an interaction term between treatment and recruitment source, an interaction term between time and treatment, an interaction term between time and recruitment source, and a three-way interaction term between time, treatment and recruitment source. Models adjusted for age, sex, race, field site, education, body mass index, and pure-tone average.
[e] Treatment effect is the estimated difference in 3-year change in the outcome comparing intervention to control.
